# Diagnostic performance and factors influencing the accuracy of EUS-FNA of pancreatic neuroendocrine neoplasms

**DOI:** 10.1007/s00535-016-1297-7

**Published:** 2016-12-24

**Authors:** Susumu Hijioka, Kazuo Hara, Nobumasa Mizuno, Nozomi Okuno, Vikram Bhatia

**Affiliations:** 1 Department of Gastroenterology, Aichi Cancer Center Hospital, 1-1 Kanokoden, Chikusa-ku, Nagoya, 464-8681 Japan; 2 Department of Gastroenterology, Fortis Escorts Liver and Digestive Institute, New Delhi, India

Dear Dr. Andoh,

We thank you for the opportunity to respond to the Letter to the Editor written by Dr. Neel Sharma regarding our recent publication entitled, “Diagnostic performance and factors influencing the accuracy of EUS-FNA of pancreatic neuroendocrine neoplasms” [1]. We agree with the opinion expressed by Dr. Sharma that operator training and skill must be factored into the findings of any study of EUS-FNA.

Skill assessment of operators would assume more relevance when only one or a few operators implement the procedures. Five expert endosonographers implemented the procedures or supervised others for the published study. We feel that having several operators negated the effects of individual skill variations, and increased the applicability of the results.

The operators are expected to develop their skills through experience accumulated through participation in the study. Two features of the study design minimized any effects on the results of baseline operator training and incremental skill gain. One was the presence of on-site pathological evaluation for all patients. The other was the division of the study period into periods I (1998–2008; the first 30 patients) and II (2009–2014; the remaining 28 patients), which would naturally include any changes in individual skill levels besides variations in equipment and mechanistic aspects of the procedures over the study period. Univariate analysis did not select the period as a significant factor.

The results for non-experts who undertake these procedures will depend on many factors including the type of lesion being sampled and the presence or absence of on-site pathological evaluation as well as other factors. We feel that trainees should be evaluated by their overall yield of EUS-FNA as they become more experienced.
